# Emerging Nanotheranostics for 5-Fluorouracil in Cancer Therapy: A Systematic Review on Efficacy, Safety, and Diagnostic Capability

**DOI:** 10.3389/fphar.2022.882704

**Published:** 2022-05-18

**Authors:** Chee Wun How, Siew Li Teoh, Jian Sheng Loh, Stella Li Kar Tan, Jhi Biau Foo, Hui Suan Ng, Stephenie Yoke Wei Wong, Yong Sze Ong

**Affiliations:** ^1^ School of Pharmacy, Monash University Malaysia, Subang Jaya, Malaysia; ^2^ School of Pharmacy, Faculty of Health and Medical Sciences, Taylor’s University, Subang Jaya, Malaysia; ^3^ Centre for Drug Discovery and Molecular Pharmacology (CDDMP), Faculty of Health and Medical Sciences, Taylor’s University, Subang Jaya, Malaysia; ^4^ China-ASEAN College of Marine Sciences, Xiamen University Malaysia, Sepang, Malaysia; ^5^ Faculty of Applied Sciences, UCSI University, Kuala Lumpur, Malaysia

**Keywords:** nanoparticle, cancer, thermal ablation, *in vivo*, tumor-targeted

## Abstract

The conventional concept of using nanocarriers to deliver chemotherapeutic drugs has advanced to accommodate additional diagnostic capability. Nanotheranostic agents (NTA), combining both treatment and diagnostic tools, are an ideal example of engineering-health integration for cancer management. Owing to the diverse materials used to construct NTAs, their safety, effectiveness, and diagnostic accuracy could vary substantially. This systematic review consolidated current NTAs incorporating 5-fluorouracil and elucidated their toxicity, anticancer efficacy, and imaging capability. Medline and Embase databases were searched up to March 18, 2022. The search, selection, and extraction were performed by the preferred reporting items for systematic reviews and meta-analysis (PRISMA) guidelines to ensure completeness and reproducibility. Original research papers involving 5-fluorouracil in the preparation of nanoparticles which reported their efficacy, toxicity, and diagnostic capability in animal cancer models were recruited. The quality of included studies was assessed using the Collaborative Approach to Meta-Analysis and Review of Animal Data from Experimental Studies (CAMARADES) checklist. Nine studies were eligible for the systematic review. There was no significant toxicity reported based on animal weight and organ histology. Complete tumor remission was observed in several animal models using chemo-thermal ablation with NTAs, proving the enhancement of 5-fluorouracil efficacy. In terms of imaging performance, the time to achieve maximum tumor image intensity correlates with the presence of targeting ligand on NTAs. The NTAs, which are composed of tumor-targeting ligands, hold promises for further development. Based on the input of current NTA research on cancer, this review proposed a checklist of parameters to recommend researchers for their future NTA testing, especially in animal cancer studies.

**Systematic Review Registration**: website, identifier registration number.

## 1 Introduction

Cancers are feared diseases due to the impact they have on individuals. Being one of the four major noncommunicable diseases emphasized by the World Health Organization, cancers alone have led to 10 million premature deaths in 2020 (Sung et al., 2021). Detection, screening, and treatment remained the current key responses toward cancers (Gates, 2014). Numerous chemotherapeutic agents have been approved for use in the past decades (Goodman et al., 1946). Antimetabolites such as 5-fluorouracil (5FU) are one of the compounds being discovered and used in combination with other consolidation therapies during World War II (Duschinsky et al., 1957). In 1980, after introducing an acceptable international standard regimen, 5FU became one of the core drugs in the regimens to treat gastrointestinal cancers (Preusser et al., 1989). However, the compound is rapidly cleared due to its hydrophilicity. The narrow therapeutic index of 5FU further prevents its administration at high doses in an attempt to prolong the availability in the blood plasma. Owing to these limitations, the nanoparticulate formulation of 5FU has been introduced to improve the pharmacokinetic properties. As a pyrimidine analogue and inhibitor of thymidylate synthase, 5FU’s dual synergy inhibition in the nucleic acids improves chemotherapeutic outcomes. To date, 5FU is still clinically relevant to treat various types of solid tumors by inhibiting the synthesis of DNA and RNA in cancer cells (Collier et al., 2020; Polat et al., 2021).

Pharmaceutical technology can modify the pharmacokinetics (PK) properties of drug molecules. One such technology involved using formulation to incorporate nanotechnology with existing medications, e.g., polymeric micelle paclitaxel (Genexol^®^) (Werner et al., 2013). These products are claimed to have improved therapeutic outcomes due to the nanoparticles’ intrinsic properties, which could lead to drug accumulation in the vicinity of tumor tissues (passive-targeting). One of the earliest pieces of evidence is the liposomal doxorubicin (Doxil^®^). Apart from the 8-folds increase in mean circulation residence time, the doxorubicin in liposomes can accumulate in the malignant exudates 10-folds more than doxorubicin alone (Gabizon et al., 1994). However, the utility of nanoparticles in drug delivery systems is not limited to treatment. A search in the literature revealed innovative nanoparticles that integrate diagnostic agents with therapeutic agents for treatment purposes, known as nanotheranostic agent (NTA) (Wang et al., 2012). These nanoparticulate systems have sizes less than 1,000 nm and are composed of diagnostic and therapeutic moieties that are either adsorbed, attached, or encapsulated within the nanostructure (Siafaka et al., 2021). Depending on the nanomaterials used, NTA could perform functions such as delivering drugs, inducting hyperthermal/photothermal effect, and imaging (Muthu et al., 2014). The approach enables tumor structure, size, and location to be assessed objectively for prognosis while the patient is still on chemotherapy.

In an ideal scenario, a multi-functional drug delivery system allows healthcare providers to monitor the cancer progression while targeting the capability of nanoparticulate treatment. Therefore, the use of contrasting agents in some oncological imaging techniques may be avoided, reducing the risk of adverse effects in already frail cancer patients. As an emerging area of interest under the umbrella of nanomedicine, the applications of NTA are highly envisioned (Chen et al., 2017). Nevertheless, its practical aspect must be validated extensively before it could merit clinical trials. Owing to the diverse materials used to construct the nanoparticles, their effectiveness, safety, and diagnostic accuracy could vary substantially. Most cancer NTAs’ review articles have focused on the introduction, preparation, and development of specific types of NTA (Roma-Rodrigues et al., 2019; Silva et al., 2019; Thorat and Bauer, 2020). But vital information such as safety, efficacy, and imaging reliability of different NTAs remained largely unknown even in the animal models. Therefore, this systematic review aimed to compare the potential of different 5-fluorouracil nanotheranostic agents (5FU-NTAs) by consolidating their *in vivo* toxicity, efficacy, and imaging capabilities. A checklist of parameters is proposed in this review to recommend the future testing of NTA in animal cancer studies.

## 2 Methods

The systematic review was performed in accordance with the principles outlined in the Cochrane Handbook for Systematic Reviews of Interventions (Higgins et al., 2022), with adaptation for preclinical studies, and reported in accordance with guidelines from the Preferred Reporting Items for Systematic Reviews and Meta-Analysis (PRISMA) (Moher et al., 2009).

### 2.1 Literature Search

The literature search was conducted from inception until March 18, 2022 using Medline and Embase databases. The keywords used were “cancer,” “theranostics,” “nanoparticle,” “*in vivo,*” and “fluorouracil” in combination with MeSH and Emtree terms.

### 2.2 Study Selection and Eligibility Criteria

Two independent reviewers (YSO and CWH) performed the abstract and title screening to include relevant research papers addressing the animal studies using NTA. Publications were included if they met all the following inclusion criteria: (1) original research paper involving 5-fluorouracil (5FU) in the preparation of nanoparticles; (2) must involve the use of an animal cancer model; and (3) have reported efficacy, toxicity, or diagnostics as the outcomes. Papers reporting only *in vitro* studies, reviews (mini, comprehensive, systematic, and meta-analyses), conference abstracts, comments, or letters to the editor were excluded. Any discrepancy between the two reviewers was resolved by discussion and consensus.

### 2.3 Data Extraction and Analysis

The following characteristic data were extracted: the types of nanoparticles with their diagnostic and targeting components, physicochemical properties such as size, PDI, zeta potential and EE of 5FU, cancer type, animal model, dose of 5FU, its frequency and route of administration. Three primary outcomes were efficacy, toxicity, and imaging properties of the 5FU-NTA.

For the toxicity and efficacy studies, the following data were extracted: animal survival in percentage and endpoint period, body weight change (%) as compared with Day 1, histological evaluation, tumor volume reduction (%) as compared with negative control and 5FU, and tumor volume change (%) as compared with Day 1. For the diagnosis study, the following data were extracted: time to maximum tumor accumulation, types of nanoparticles, targeting moiety or mechanism, end-point tissues distribution, and imaging method.

### 2.4 Quality Assessment

The Collaborative Approach to Meta-Analysis and Review of Animal Data from Experimental Studies (CAMARADES) checklist was used to assess the study quality (Higgins et al., 2022). The tool contained 14 questions that assessed if there was a bias in the study design. With the highest score of 14, the higher score assessed for the study indicated a better methodological quality of the study (Macleod et al., 2004).

## 3 Results

### 3.1 Study Selection

**FIGURE 1:**
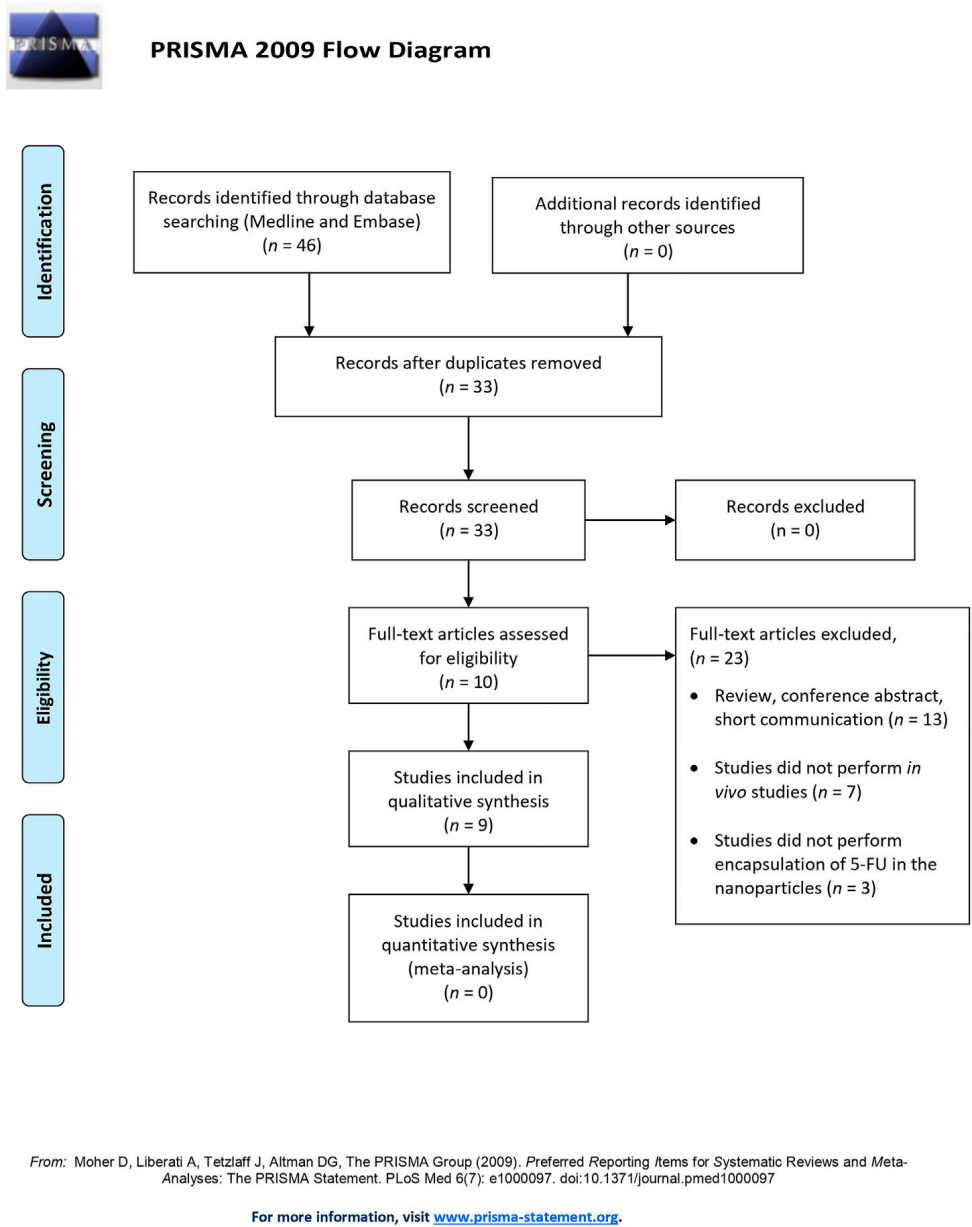
The Preferred Reporting Items for Systematic Reviews and Meta-Analysis (PRISMA) flow diagram of study selection.

The initial search of the online database yielded 46 potential articles, among which 13 duplicates were removed. Of the remaining studies screened, only 33 full-text articles were reviewed according to the inclusion and exclusion criteria. The excluded studies were those that did not perform *in vivo* studies (*n* = 7), b) use 5FU as the chemotherapeutic agent (*n* = 3), or c) conduct original research (*n* = 13). As a result, nine studies were included in the qualitative analysis. Figure 1 illustrates the flow of study selection.

### 3.2 Study Characteristics

The characteristics of the studies are summarized in Table 1. Almost half of the studies (*n* = 4, 44.4%) were conducted in Iran, followed by China (*n* = 2, 22.2%), the United Kingdom (*n* = 1, 11.1%), Australia (*n* = 1, 11.1%), and South Korea (*n* = 1, 11.1%). Almost half of the studies (*n* = 4, 44.4%) developed NTA using inorganic materials, in which gold nanoparticle is the majority (*n* = 3, 33.3%). Another 33.3 (*n* = 3) of the studies developed the NTA using the hybrid of organic and inorganic materials. Only two studies used organic material to achieve nanotheranostic properties without using transition metals. 66.7% of the studies (*n* = 6) did not report the essential physicochemical characteristics, such as nanoparticle size, polydispersity index (PDI), zeta potential, and entrapment efficiency (EE) of 5FU.

**TABLE 1:** General characteristics of the included studies.

Study (year)	Country	Type of nanoparticle	Diagnostic agent	Targeting agent	Size (nm)∼	PDI	ζ-Potential (mV)∼	EE of 5FU (%)	Type of cancer	Animal model/Cancer cell	Unit dose of 5FU (PA) (mg/kg)	Frequency	ROA	Outcomes	Study quality
Conde et al. (2015)	United Kingdom.	Gold	Gold	Hairpin-DNA-Q705 (mRNA)	30.9 ± 0.5 (DLS)	NR	NR	NR	Breast	Female SCID hairless congenic mice, MDA-MB-231	0.65	1	IT	Efficacy, toxicity, imaging	6
Babaei et al. (2017)	Iran	Gold-silica	Gold	EpCAM	63.12 (DLS)	0.21	+8.4	23.87	Liver	C57BL/6 nude mice, HepG2^b^	0.012	1	IV	Imaging	6
Fang et al. (2017)	China	Gold	Gold	NR	Length 54.14 ± 4.39, width 15.87 ± 1.28^a^ (TEM)	NR	−3 ± 2.54	29.1	Skin (Melanoma)	Female BALB/c nude mice, A375	1.455	2	IV	Efficacy, imaging	4
Wu et al. (2017)	China	Dendrimer	Squaraine dye	Substance P	20–110 (DLS and TEM)	NR	NR	16	Lung	Female BALB/c nude mice, A549	0.39	8	IV	Efficacy, toxicity, imaging	5
Mohammadi Gazestani et al. (2018)	Iran	Graphene Oxide-SPION	Fe_3_O_4_	Fe_3_O_4_	72.9 (DLS); 15–20 (TEM)	NR	-30.82 ± 0.57	50	Colon	Male BALB/c mice, CT26	3	1	IV	Efficacy, imaging	7
Li et al. (2018)	Australia	Layered double hydroxide	Copper	NR	41.2 ± 5.4 (DLS)	NR	NR	NR	Colon	Female BALB/c nude mice, HCT116	2	2	IV	Efficacy, toxicity, imaging	6
Samiei Foroushani et al. (2019)	Iran	Graphene oxide	DTPA-Mn (II)	Folic acid	70–180+	NR	NR	NR	Colon	Female BALB/c mice, CT-26	1.2	7	IV	Efficacy, toxicity, imaging	5
Nejadshafiee et al. (2019)	Iran	Bio-Metal-Organic Framework	Fe_3_O_4_	Folic acid -chitosan conjugate	90 (TEM); 128 (DLS)	0.21	+5.31	60	Breast	BALB/c mice, M109^b^	NR	NR	IV	Imaging	3
Lee et al. (2020)	South Korea	Human serum albumin	Cy7 fluorophore	B5 peptide	208.2 (DLS)	0.21	-12.9	NR	Colon	Male BALB/c nude mice, human colorectal cancer fragment	2	5	IV	Efficacy, Toxicity, Imaging	7

Abbreviations: 5FU, 5-Fluorouracil; DLS, dynamic light scattering; EE, encapsulation efficiency; IV, intravenous; IT, intratumoral; NR, not reported; PA, per administration; PDI, polydispersity index; ROA, route of administration; SPION, superparamagnetic iron oxide nanoparticles; TEM, transmission electron microscopy.

a Rod shape.

b Sex of animal is not mentioned.

+ size assumed based on same carrier with different drug.

∼ All values are mean ± standard deviation (SD).

### 3.3 Toxicity, Efficacy, and Diagnostic Imaging of 5FU-NTA

Seven of the nine studies conducted antitumor efficacy tests in which only five of them performed toxicity testing by measuring the body weight change (*n* = 4, 44.4%) and histological examination (*n* = 1, 11.1%). All the studies (*n* = 9, 100%) reported the use of various types of biomedical imaging methods, i.e., magnetic resonance imaging (MRI), near-infrared fluorescence imaging (NIRF), and computerized tomography (CT) scan.

### 3.4 Efficacy

#### 3.4.1 Mode of Treatment

Out of the seven studies, the 5FU-NTA were found to be functionalized to illicit various modes of treatments (Sung et al., 2021) 5FU-NTA coupled with active targeting only (*n* = 3, 50.0%) (Gates, 2014), 5FU-NTA coupled with thermal ablation only (*n* = 2, 33.3%), and (Goodman et al., 1946) combination of both (Sung et al., 2021) and (Gates, 2014) (*n* = 2, 22.2%) (Table 2). Overall, these functionalized NTA were more efficacious (over 40% tumor volume reduction as compared with negative control) than the nonfunctionalized nanoparticles that delivered 5FU only (less than 25% tumor volume reduction in comparison with negative control). Two studies were able to achieve a complete tumor remission via hyperthermic treatment, while one study reported a partial tumor remission (50% remission) (*n* = 1, 16.7%) via an active-targeting strategy. Based on the tumor volume reduction, the overall efficacy of 5FU-NTA was the highest when combined with hyperthermal therapy, followed by tumor-targeted NTA and nonfunctionalized nanoparticles. The efficacy of free 5FU has been enhanced with the use of nanoparticles in all studies except Conde et al. (2015), which demonstrated that free 5FU was more efficacious than encapsulated 5FU with 21% tumor volume reduction in tumor-bearing SCID hairless congenic mice (Conde et al., 2015).

**TABLE 2:** Efficacy and toxicity of nanoparticles encapsulated/conjugated with 5FU.

Authors^a^	Fang et al. (2017)	Li et al. (2018)	Conde et al. (2015)	Lee et al. (2020)	Mohammadi Gazestani et al. (2018)	Samiei Foroushani et al. (2019)	Wu et al. (2017)	Mohammadi Gazestani et al. (2018)	Li et al. (2018)	Conde et al. (2015)
**Variables**										
Mode of Treatment	Photothermal therapy	Photothermal therapy	Active targeting and mRNA	Active targeting and irradiation	Photothermal and magnetic targeting	Active targeting	Active targeting	Magnetic targeting	NA	NA
Total dose of 5FU (mg kg^−1^)	2.91	4.00	0.65	10	3.00	8.40	3.12	3.00	4.00	0.65
Type of NP	IO	H	IO	O	H	H	O	H	H	IO
**Tumor volume reduction (%)**										
Compare to negative control	100	100	95	87.5	85	81	58.1	40	25	12
Compare to free 5-FU	100	NA	93	83.3	85	58	35.7	40	NA	+21
**Tumor volume change (%)^+^**	-100 (CR)	-100 (CR)	-50 (PR)	+150	+600	+1,000	+900	+3,000	+800	+417
**Survival (%, endpoint by days and days difference)**										
Compare to negative control	NR	100%, 24, +8	NR	NR	100%, 40, +16	NR	NR	0%, 40, +14	0%, 24, +8	NR
Compare to free 5FU	NR	NR	NR	NR	100%, 40, +11	NR	NR	0%, 40, +11	NR	NR
**Body weight change (%)^+^**	NR	+7.1	+6.7	+8	NR	NR	+5.7	NR	+15.4	+16.1
**Histological examination**	NA	NA	NA	NA	NA	Y	NA	NA	NA	NA

Abbreviations: 5FU, 5-fluorouracil; NP, nanoparticle; IO, inorganic; H, hybrid; O, organic, NA, not available; NS, not significant; NR, not reported; CR, complete remission; PR, partial remission.

a Arranged according to the descending order of tumor volume reduction (%).

^+^ As compared to tumor size on Day 1. A negative sign means reduction while positive sign means increment.

#### 3.4.2 Total Dose of 5FU

The treatments in all studies were administered parenterally to assume maximum bioavailability in the tumor-bearing animals. The dose of 5FU used for NTA in anticancer studies ranged from 0.56 to 10 mg/kg. Although the range of dose varied 18 times, it did not seem to affect the treatment efficacy. This could be observed in the studies by Conde et al. (2015) and Foroushani et al. (2019), whereby the former reported a partial tumor remission at a dose of 0.65 mg kg^−1^ while the latter inhibited only 19% of tumor volume growth at a higher dose of 8.40 mg kg^−1^ (Conde et al., 2015). Additionally, administering a similar dose may not elicit the same therapeutic outcomes. Fang et al. (2017) and Mohammadi Gazestani et al. (2018) have both administered ∼3 mg kg^−1^ of 5FU into tumor-bearing animals, but the outcome varied from complete tumor remission (100% tumor volume reduction) to growth inhibition (15% tumor volume reduction) (Fang et al., 2017; Mohammadi Gazestani et al., 2018). Since all studies (*n* = 7) reported only one concentration (unit dose of 5FU) for their animal testing, the effect of concentration on tumor response could not be established even by the inter-study data. It is worth noting that the observed data from the shortlisted studies should be interpreted cautiously because the dose of the drug is inherently determined by various factors, including the type of cancer, vehicle, route of administration, etc. Section 4.6 further elaborates on other factors that could affect the outcome of this study.

#### 3.4.3 Type of Material

The choice of materials was found to have impacted the physicochemical properties of NTA, which in turn alter their biodistribution, clearance, toxicity, and effectiveness of the incorporated drugs. The majority of the studies used a hybrid of organic and inorganic materials (*n* = 3), followed by fully inorganic (*n* = 2, 28.5%) and organic (*n* = 2, 28.5%) materials. The type of material used depends mainly on the mode of treatment. Metal-based components such as superparamagnetic iron oxide nanoparticles (SPION), gold, and manganese are the materials of choice when magnetic resonance imaging (MRI) or computed tomography scan (CT) was the imaging method. With appropriate reporting molecules such as squaraine dye (Wu et al., 2017) and Cy7 fluorophore (Lee et al., 2020), metal- and organic-based nanomaterials are compatible with the fluorescence imaging method. It is noteworthy that organic-based materials are not suitable for MRI or CT scans.

### 3.5 Toxicity

**FIGURE 2:**
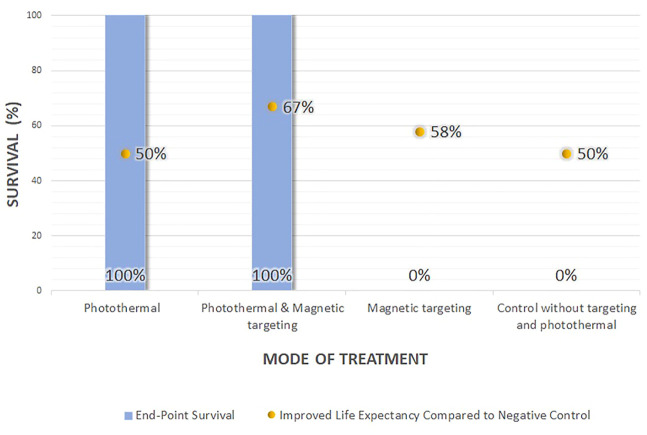
The effect of treatment method on the survival rate of rodents.

The survival data (the survival rate and life extension percentage), change in the body weight, and histological examination could provide information on potential NTA toxicity (Table 2). Out of the six efficacy studies, only 22.2% of the studies (*n* = 2) reported on the survival data, 66.7% (*n* = 4) reported the body weight change, and 16.7% (*n* = 1) provide histological examination. Based on the reported data, the NTAs, composed of either organic, inorganic, or hybrid materials, are relatively innocuous at the administered dose and are highly biocompatible (Conde et al., 2015; Wu et al., 2017; Li et al., 2018). In survival data, only one study compared the survival of NTA treated group to both negative control and the free drug-treated group, while the other studies compared the survival to the negative control. All the NTAs (either photothermal or a combination of photothermal and magnetic targeting) improved the survival rate up to 100% and life expectancy to more than 50% compared with the negative control (Figure 2). Even though the application of magnetic-targeting did not improve the survival rate in Mohammadi Gazestani et al. (2018), it increased life expectancy to 58 and 46% compared with negative control and free control 5-FU, respectively.

All NTAs did not result in significant toxicity based on the increment in the animal’s body weights throughout the test period. Instead, an increased body weight ranging from 6.7 to 16.1% was observed. No significant weight reduction was detected in the tumor-bearing mice that received no treatment at all. This precludes the interference of the tumor burden has on the recorded weights. Foroushani et al. (2019) performed histological evaluation using H&E staining on the liver, spleen, lung, kidney, brain, and bone marrow of the treated mice, showing no sign of toxicity (Samiei Foroushani et al., 2019).

### 3.6 Imaging Method

All diagnostic functionality of the NTAs (*n* = 8) was demonstrated using various tumor imaging techniques in tumor-bearing animals (Table 3). In terms of the imaging techniques, MRI was the most frequently used (*n* = 4, 50%) and NIRF (*n* = 4, 50%), followed by CT scan (*n* = 1, 12.5%). 71.4% of the studies (*n* = 5) evaluated the imaging performance of tumor-targeted NTAs. However, only 28.6% of them (*n* = 2) compared their imaging performance to the nontargeted ones (Babaei et al., 2017; Fang et al., 2017). While most of the studies (*n* = 4, 57.1%) used ligands to achieve a tumor-targeting mechanism, only 1 (14.3%) study utilized external magnetic fields to alter the NTA’s distribution in the body.

**TABLE 3:** Relative time to achieve maximum tumor accumulation of 5FU encapsulated/conjugated nanotheranostics and their imaging techniques.

Time to maximum tumor accumulation	Types of nanoparticles	Targeting moiety/mechanism	End-point tissues distribution	Imaging method
1 h	Fe_3_O_4_@Bio-MOF-FC Nejadshafiee et al. (2019)	Folic acid/Folate receptors	NR	Magnetic Resonance
2 h	Fe_3_O_4_-PLGA GO (Mohammadi Gazestani et al., 2018)	SPION/magnetism	Infrared thermal imaging recorded higher heat at tumor region than whole body	Magnetic resonance
3 h	Folate-graphene manganese Samiei Foroushani et al. (2019)	Folic acid/folate receptors	tumor > spleen > liver > kidney (Performed with ICP-OES, 24 h)	Magnetic resonance
6 h	EpCAM-PEG-AU-rhodamine Babaei et al. (2017)	EpCAM/Cell adhesion molecules	6 h - abdominal region, 24 h - bladder region	Fluorescence/CT scan
	PEG-AU-rhodamine Babaei et al. (2017)^b^	NA	6 h - chest region, 24 h - no significant accumulation	Fluorescence/CT scan
24 h	P-FU4 Wu et al. (2017)^a^	Substance P/Neurokinin-1 receptors	Liver > tumor > spleen > kidney; whereas, heart, lung, stomach are unobservable	Fluorescence (near infrared)
	GN-ICG NP Fang et al. (2017)^a^	NA	Tumor > liver > kidney > lung > heart > spleen	Fluorescence (near infrared)
	Cu-LDH NP Li et al. (2018)^a^	NA	Thermal imaging recorded higher heat at tumor region than whole body	Magnetic resonance
48 h	Cy7-B5-HSA Lee et al. (2020)	B5 peptide/LRP-1 receptor	Undetected in other organs	Fluorescence (near infrared)
Undetectable	ICG-NHS Fang et al. (2017)	NA	Liver > spleen > lung > kidney > bladder	Fluorescence (near infrared)

Abbreviation: Cy7-B5-HSA, Cy7-B5 peptide coupled human serum albumin; Fe_3_O_4_Bio-MOF-FC, Fe_3_O_4_ magnetic Bio-Metal-Organic Framework coated with folic acid-chitosan; Fe_3_O_4_-PLGA GO, polylactic-glycolic acid magnetite nanographene oxide; EpCAM-PEG-AU-rhodamine, epithelial-cell adhesion molecule-polyethylene glycol-gold loaded with rhodamine; ICP-OES, inductively coupled plasma-optical emission spectrometry; P-FU4, substances P-squarine-coated fluorouracil dendrimer.

a Performed tumor accumulation monitoring for more than 3 time points.

b Low intensity compared with NTA, with targeting moiety (EpCAM-PEG-AU-rhodamine).

**FIGURE 3:**
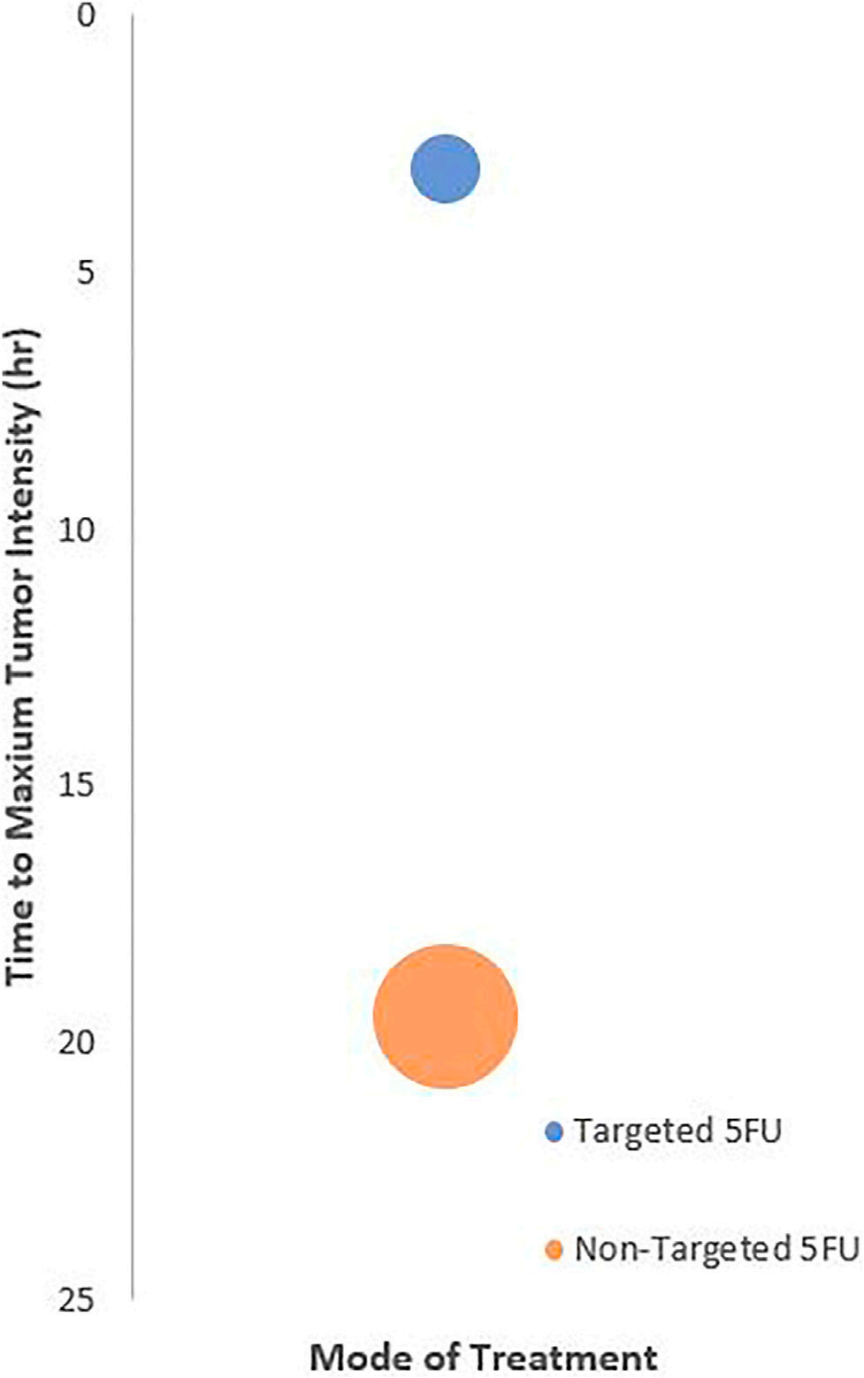
Relationship between treatment targetability and the time to achieve maximum tumor imaging intensity. The center of bubble plot represents the average time acquired from the samples of all extracted studies (n = 4). The radius indicates standard deviation time.

All NTAs were administered intravenously to the animals. NTAs that were tagged with targeting moiety seemed to achieve maximum tumor accumulation faster (within 6 h) than those without it (Figure 3). These results were reflected qualitatively and quantitatively using image contour in all studies (*n* = 8) and signal intensity (*n* = 2, 25%), respectively. In terms of biodistribution, all the NTAs (*n* = 8) have shown preferential accumulation in the tumor region, followed by the major organs responsible for drug elimination, such as the liver and kidney.

### 3.7 Quality Assessment

The overall quality score based on the CAMARADES tool ranged from 3 to 7, out of 14, with a median score of 6 (Table 4). All studies (*n* = 9) were published in peer-reviewed journals using rodents as cancer models. Majority of the studies reported on the statements of compliance with regulatory requirements (*n* = 8, 88.9%), study funding (*n* = 8, 88.9%), and possible conflict of interest (*n* = 6, 66.7%). All studies did not report the allocation concealment, blinded assessment of outcome, sample size calculation, prespecified inclusion and exclusion criteria, and animals excluded from the analysis. There was no clear correlation between study quality and the main outcomes (efficacy, toxicity, and imaging properties).

**TABLE 4:** Quality assessment using CAMARADES checklist.

No	Criteria	Mohammadi Gazestani et al. (2018)	Lee et al. (2020)	Li et al. (2018)	Babaei et al. (2017)	Conde et al. (2015)	Samiei Foroushani et al. (2019)	Wu et al. (2017)	Fang et al. (2017)	Nejadshafiee et al. (2019)
1	Publication in peer-reviewed journal	Y	Y	Y	Y	Y	Y	Y	Y	Y
2	Statement of control of temperature	Y	NM	NM	NM	NM	Y	NM	NM	NM
3	Randomization to treatment and control	Y	Y	NM	NM	NM	NM	NM	NM	NM
4	Allocation concealment	NM	NM	NM	NM	NM	NM	NM	NM	NM
5	Blinded assessment of outcome	NM	NM	NM	NM	NM	NM	NM	NM	NM
6	Avoidance of intrinsically neuroprotective anesthetics	NM	NM	NM	NM	NM	NM	Y	NM	NM
7	Use of animals with cancer	Y	Y	Y	Y	Y	Y	Y	Y	Y
8	Sample size calculation	NM	NM	NM	NM	NM	NM	NM	NM	NM
9	Statement of compliance with regulatory requirements	Y	Y	Y	Y	Y	Y	Y	Y	NM
10	Statement regarding possible conflict of interest	Y	Y	Y	Y	Y	Y	NM	NM	NM
11	Physiological monitoring	NM	Y	Y	Y	Y	NM	NM	NM	NM
12	Prespecified inclusion and exclusion criteria	NM	NM	NM	NM	NM	NM	NM	NM	NM
13	Reporting animals excluded from analysis	NM	NM	NM	NM	NM	NM	NM	NM	NM
14	Reporting of study funding	Y	Y	Y	Y	Y	NM	Y	Y	Y
	Total score (out of 14)	7	7	6	6	6	5	5	4	3

Abbreviations: Y, yes; NM, not mentioned

### 3.8 Proposed Checklist

Guidelines pertaining to laboratory animal care and use (National Research Council (US), 2011), development of animal tumor models, and anticancer drugs dosage regimens have long been established (Gurney, 2002; Workman et al., 2010). Nevertheless, to the best of our knowledge, there is no guideline on the development and efficacy testing of nanoparticles in animal cancer models. Based on the best practices and flaws in the reported outcomes, we proposed the Preclinical *in vivo* Cancer Nanotheranostics Tool (PICANT) checklist to assist in the refinement of study design for nanoparticle use (particularly NTA) to ensure the results reliability on animal cancer models (Table 5). This checklist aimed to support researchers in designing their study protocol, assist manuscript reviewers in critically assessing the study quality and as guidelines for institutions in the decision-making process.

**TABLE 5:** PICANT checklist.

Checklist items	Descriptions
(A) physicochemical properties of nanoparticle	
Material	Nanomaterials used and categories based on element
Size range (technique)	Sizing information presented with sizing techniques
Charges	Surface charges or zeta potentials
Drug content	Quantification of drug amount in the nanoparticles
Stability	Drug release profile under storage condition
(B) Antitumoral effectiveness and toxicity	
Animal species	The type of animal used for cancer model
Tumor volume/weight	Change of volume/weight compared to Day 1
Dose and dosing frequency	The normalized amount of drug comparable to control
Route of administration	The route of nanomaterials administered into the animal
Control groups	Free-drug and negative controls
Survival	Survival time and study endpoint
General Toxicity profile	Body weight change
Organ toxicity profile	Histology of major organs, biochemical tests
Blood toxicity	Blood profile, biochemical tests
(C) Imaging for diagnostic/prognostic purpose (if applicable)	
Imaging technique	Biomedical imaging use to study tumor
Reporting probe	Molecule that produces imaging signals
Control groups Imaging	Comparison of between functionalized and nonfunctionalized nanoparticles
Intensity-to-noise	A ratio that can relate to image quality
Localization	Organ images other than tumor for information on biodistribution
Time interval	Time range where image acquisition is made

The PICANT checklist is developed based on the use of NTA in animal cancer studies. Rational modification on the checklist is recommended for fitting different study designs that utilize nanoparticles.

## 4 Discussion

To the best of our knowledge, this is the first review that systematically summarized the potential use of different 5FU-NTAs in animal tumor models by consolidating the main outcomes in terms of treatment toxicity, antitumor efficacy, and imaging capabilities. The materials used to prepare the core of NTAs can generally be classified as organic (e.g., dendrimer), inorganic (e.g., gold, copper, SPION), or hybrid. Various targeting ligands can be incorporated into the 5FU-NTAs to achieve target-specific delivery. 5FU-NTAs coupled with thermal tumor ablation has shown potentially higher antitumor activity than conventional 5FU nanoparticles or free 5FU in animal tumor models. Standardized protocols and data related to the PK and toxicities of NTA are recommended to interpret the efficacy data better. A checklist of parameters has also been proposed in this review for guidance in future NTAs animal cancer studies (Table 5).

### 4.1 NTAs as Potential Delivery Systems for 5FU

The basic compositions of NTAs include a nanosized carrier, drug, and imaging probe. Depending on the materials used to form the matrices of the carriers, the NTA can be categorized into organic, inorganic, or a combination of both. Data showed that the inorganic nanoparticles, particularly gold and iron oxide, were the most preferred nanomaterials in the NTA cancer studies.

Gold nanoparticles are extensively studied as a drug delivery system for their innate ability to absorb near-infrared radiation and induce a hyperthermal effect. Thermal tumor ablation therapy, which kills cancerous cells using elevated heat, is routinely performed in clinical settings because of its cost-effectiveness and less traumatic compared with surgery (Ashikbayeva et al., 2019). Owing to the unique electronic configuration, gold nanoparticles are a good image contrasting agent with a high X-ray attenuation coefficient for imaging modalities such as CT and MRI (Mahan and Doiron, 2018). Although gold nanoparticles are chemically resistant to oxidation, their colloidal state requires stringent storage conditions to prevent aggregations. While there are two gold nanoparticle-based treatments currently in clinical trials (NCT03020017 and NCT00848042) (Anselmo and Mitragotri, 2019), none has been approved by the Food and Drug Administration to date (Bloise et al., 2020). With the lack of human data, there are still concerns such as toxicity and PK involving the distribution, metabolism, and excretion of gold nanoparticles that might further impact their clinical use.

Mohammadi Gazestani et al. (2018) and Nejadshafiee et al. (2019) utilized iron oxide nanoparticles as the NTAs to deliver 5FU. This material can be induced to perform thermal tumor ablation, similar to gold nanoparticles (Ashikbayeva et al., 2019). Owing to their high magnetic moment density, iron oxide nanoparticles can be artificially maneuvered by an external magnetic field to concentrate chemotherapeutic drugs in tumor tissues (Mohammadi Gazestani et al., 2018).

All NTAs in the recorded study were either inorganic or hybrid nanomaterials except Wu et al. (2017) and Lee et al. (2020). Wu et al. assembled dendrimers that encapsulated 5FU as the drug model, substance P as the active targeting agent, and squaraine dye as the imaging fluorophores (Wu et al., 2017). Meanwhile, Lee et al. incorporated Cy7 fluorophore as the imaging entity and B5 peptide as the active targeting agent with human serum albumin (Lee et al., 2020). The nanomaterials in most of the 5FU-NTA studies were transition metals due to their widely existing use as contrasting agents. In contrast, organic materials do not share the same property as transition metals. Therefore, the development of organic-based imaging nanoparticles may require extra steps of conjugation for the image reporting molecules. In other words, extensive stability and structural validation are required before being formulated with drugs. In the following section, the comparison of the various nanocarriers’ efficacy, toxicity, and diagnostic properties was discussed.

5FU is one of the core drugs used to treat solid tumors. Being hydrophilic in nature, 5FU is rapidly cleared from the body with a half-life of 8–20 min (Diasio and Harris, 1989). Nevertheless, the administration of 5FU at higher doses to prolong the short availability in the systemic circulation was not feasible due to its narrow therapeutic window. This has led to the modest clinical activity of 5FU at standard doses. Adherence to therapeutic drug monitoring is highly recommended to maintain the plasma concentration at 450–550 μg L^−1^ (Gamelin et al., 2008). In the recorded studies, the antitumor effect of 5FU was remarkably inferior even to be claimed as a standard chemotherapeutic drug. This can be seen in the tumor volume treated with free 5FU that seemed to be on par, if not worse, with those without treatment at all (Mohammadi Gazestani et al., 2018). The treatment of free 5FU from 0.65 to 4.00 mg kg^−1^ (human equivalent dose of 0.05–0.32 mg kg^−1^) (Nair and Jacob, 2016) could not achieve a complete tumor remission (with less than 25% of tumor volume reduction as compared with negative control) (Conde et al., 2015; Li et al., 2018). The dose selected is much lower than the actual dose of 5FU used clinically, which is 10–20 mg kg^−1^ for 5–10 days intravenously (Zhang et al., 2018).

The dosing frequency selected by the authors varied substantially from 2 to 8 doses over the entire course of the experiment (Fang et al., 2017; Wu et al., 2017). The extracted studies with a quality score higher than the median (>6 out of 14) tend to have higher dosing frequency, in line with the clinical practice where 5FU is given in the course of several cycles. The dose selected varied significantly among the animals of the same species, such as BALB/c mice (Wu et al., 2017; Mohammadi Gazestani et al., 2018; Samiei Foroushani et al., 2019). However, no specific reason was provided to rationalize the choice of dose for all the studies. To compare the effectiveness of 5FU-NTA and free 5FU, an equivalent amount of 5FU needs to be established *via* calculations using encapsulation or loading efficiency. Unfortunately, these data were also not reported in Conde et al. (2015), Li et al. (2018), and Samiei Foroushani et al. (2019). As the exact amount of drug being encapsulated/adsorbed was not known, the antitumor effect of the nanoparticles could not be correlated to the stated dose with high confidence.

### 4.2 Various Treatment Strategies of 5FU-NTAs

Apart from the 5FU being incorporated as a chemotherapeutic drug into the NTAs, some NTAs are tailored to deliver extra treatment strategies. A consolidation of the recorded data revealed a clear association between the antitumoral activity of 5FU-NTA and the treatment strategy. The efficacy of 5FU at an equivalent dose was greatly improved by the additional approach such as photothermal or tumor-targeting (Conde et al., 2015; Li et al., 2018). In particular, the 5FU-NTA designed by Li et al. (2018) achieved complete tumor remission with 100% survival using a hybrid type of materials to perform the dual functions. In a separate study, Fang et al. (2017) also demonstrated a similar result in tumor-bearing mice using gold nanorod (inorganic) to elicit a hyperthermal effect. On the other hand, although tumor-targeted strategy could also enhance the antitumoral activity of 5FU-NTA, they are relatively less effective than thermal ablation, with an average tumor growth inhibition between 58.1 and 87.5% (Wu et al., 2017; Samiei Foroushani et al., 2019). The use of ligands or magnet on 5FU-NTA to actively target tumors has proven to increase 5FU concentration in the vicinity of the tumor. However, the active component that kills cancer cells mainly relies on the cytotoxic property of 5FU. Unlike the tumor-targeted strategy, the hyperthermal effect induced by the thermal-ablation strategy did not only increase the membrane penetration of 5FU-NTA but also caused apoptosis or ferroptosis in the cancer cells. It is postulated that this multimodal action could be the reason for the complete tumor remission in Li and Fang’s studies.

The attempt to integrate both the hyperthermal and tumor-targeted strategies into 5FU-NTA did not result in greater antitumoral performance than using the hyperthermal approach alone (Mohammadi Gazestani et al., 2018). Nevertheless, it is noteworthy that both Li et al. (2018) and Fang et al. (2017) applied hyperthermal treatment twice to achieve remarkable outcomes, while Mohammadi Gazestani et al. (2018) only applied it once. It is suggested that the 5FU-NTA with functionalized treatment, regardless of single or multi-modes, enhanced the efficacy of 5FU in the animal cancer models. On top of that, the tumor thermal ablation 5FU-NTA was shown to be the most effective treatment.

### 4.3 Safety Concerns

The safety of nanomedicine has been a major dispute in the drug development process not only for humans but for the environment as well (Xin et al., 2021). However, animal models remain the bedrock of biological and toxicological research in the early development phases. In the context of nanotoxicology, the manipulation of materials at nanosize levels notably changes the way how it interacts with living organisms and the environment, especially the inorganic nanomaterials (Patel and Shah, 2017). In our extracted data, inorganic and hybrid materials were popular choices for NTA due to their biomedical imaging capability, such as gold and iron oxide nanoparticles. However, their usage in the scientific community has always been debatable as these nanomaterials are not biodegradable and have a long retention time in the body. For instance, a toxic response was observed in the liver, lungs, and kidneys of animals after being treated with 0.8 mg kg^−1^ of iron oxide nanoparticles (Lewinski et al., 2008). Meanwhile, Mohammadi Gazestani et al. (2018) administered the same iron oxide nanoparticle, which was 11-folds (∼9 mg kg^−1^) higher than the mentioned dose for their 5FU-NTA. Therefore, there is a concern on the long-term toxicities of the NTA used. Our data showed that not all studies reported basic toxicity profiles such as animal mortality data, body weight change, and histological examination. The lack of toxicological information does not only affect the verification of the toxic effects, but also hampers the toxicity comparison between different types of 5FU-NTA.

The dose of 5FU is limited by its narrow safety profile. At therapeutic dose, incidences of gastrointestinal, cardiovascular, neuro-toxicities, and myelosuppression were observed in patients (Fang et al., 2016). In fact, 49.1–61.0% of the patients receiving 5FU require dose adjustment at some point during their treatment cycles (Morawska et al., 2018). Relevant to this clinical information, data on blood profile, biochemical tests, and organ histopathology should be incorporated into the 5FU-NTA study design to verify information on organs damage. In Li et al. (2018), Conde et al. (2015), and Wu et al. (2017) studies, the “non-toxic” claims solely based on an increase in body weight could be inconclusive according to the Organization for Economic Co-operation and Development (OECD) guidelines.

Apart from that, PK and biodistribution data are informative to predict the potential adverse effects of NTAs in clinical and diagnostic applications. None of the work addressed PK in their study except Wu and others, who developed a dendrimer-based NTA (p-FU4) (Wu et al., 2017). Despite having only 5 data points and no positive control in their PK study design, the results indicated that 5FU’s short half-life was not altered by nanoparticles, i.e., about 20 min (Diasio and Harris, 1989). The measurement of plasma p-FU4 concentration was feasible as the blood was sampled at a predetermined time. Here, the concern over premature dissociation of 5FU that would further interfere with the measurement accuracy was minimal since the composition of p-FU4 was held entirely by amide bonds. Compared with mere physisorption in Mohammadi Gazestani’s 5FU-graphene oxide nanoparticles, there was a phenomenal burst release of 5FU-NTA in the first few hours when incubated at 37°C (Mohammadi Gazestani et al., 2018). Owing to the presence of 5FU in the plasma in free form and nanoparticle-incorporated form, reliable measurement to exclusively identify the drug amount with minimal errors might not be achievable. This further complicates the data interpretation for the PK study.

For successful clinical translation, the toxicity data have a high inferential value for evaluation by regulatory agencies. While many potential anticancer agents offer promise for cancer patients, the toxicity data, particularly survival, is the trade-off point that might drive the decision of treatment choice (Wong et al., 2013). Therefore, researchers should emphasize the investigation of toxicity and PK on the top of the efficacy of treatment.

### 4.4 Imaging Modalities

Biomedical imaging plays a pivotal role in the diagnosis of diseases and disorders. As one of the main pillars of comprehensive cancer care, imaging data are indispensable in supporting cancer screening, staging, and treatment. All the selected 5FU-NTAs assessed tumor imaging via various techniques, i.e., MRI, CT, and NIRF. Owing to the nature of these different techniques, a direct comparison of image quality cannot be achieved. Instead, the time required to attain the respective optimal image intensity was measured. Regardless of the imaging technique used in the studies, the time for NTA to achieve optimal imaging intensity was correlated to the presence of targeting moieties on the NTAs.

A rapid and clear image of the tumor could be acquired when tumor-targeted strategies were used. One of the studies exploited the overexpressed protein on cancerous cells, i.e., folate receptors. The idea of using folic acid (FA) as a homing-ligand for cancers targeting has been well established due to its receptor being over-expressed in epithelial, ovarian, cervical, breast, lung, kidney, colorectal, and brain tumors (Fernández et al., 2018). With a molecular weight of 441 Da, FA is stable over a broad range of pH and temperature, inexpensive and non-immunogenic in nature. The ability to retain its affinity toward folate receptors even after conjugation with other molecules makes it highly relevant for cancer-targeting purposes. In another report, Mohammadi Gazestani and others successfully demonstrated the magnetism-guided tumor-targeting mechanism by selectively applying an external magnet to 1 side of the tumor in the rodent (Mohammadi Gazestani et al., 2018). Instead of allowing the body to dictate the fate of the NTA, this finding could offer significant enhancement to visualize the internal structure of the tumor and a more reliable way to manipulate the NTA biodistribution.

The main types of imaging used in modern medicines are radiography (film and CT), MRI, ultrasound, and positron emission tomography (PET). Only CT, MRI, and PET can offer 3-dimensional imaging of tumors. Coherent to clinical practices, the popular imaging techniques for NTA studies are MRI and CT (Li et al., 2018; Mohammadi Gazestani et al., 2018; Nejadshafiee et al., 2019; Samiei Foroushani et al., 2019). Although NIRF has been proposed in the healthcare system for more than 2 decades, it has not been used as a standard cancer diagnostic tool (Chen et al., 2015). One of the main reasons is its poor tissue penetration (<1 cm) caused by the background light scattering. In small animals, this drawback does not hinder its utility for cancer imaging (Fang et al., 2017; Wu et al., 2017). In humans, however, the inability to monitor greater tissue depth has restricted the usage of NIRF probes to only superficial imaging and surgical guidance (Kosaka et al., 2009).

Rhodamines, cyanine dyes, and coumarins are commonly used fluorescence probes in clinical settings. Fang et al. (2017) designed the NTA coupled indocyanine (fluorescent probe) to silica-coated gold nanorods for detecting melanoma cells. As an approved probe for ophthalmic angiography and to evaluate cardiac output, the choice of using indocyanine seemed to achieve a desirable tumor monitoring purpose in the mice model, and it is particularly relevant to primary melanoma since it is a superficial tissue. Unlike these tissues, the probe was ineffective for imaging slightly deeper ones, even for the lymph nodes. A study conducted by Namikawa’s group has highlighted indocyanine’s limitation in visualizing sentinel lymph nodes around a cutaneous melanoma, especially in patients with a high body mass index (Namikawa et al., 2014). Because of its poor tissue penetration, noninvasive NIRF imaging of tumors in deep organs (such as liver, lung, and brain) is currently unrealistic. There is still considerable room for improvement in the sensitivity of the fluorophore in terms of image quality and brightness (quantum efficiency). Although NIRF technology can compensate for some limitations of conventional imaging modalities, its clinical relevance can only be reliable when combined with other imaging modalities or applications.

### 4.5 Quality Assessment

Quality assessment was performed on all studies to examine the confidence of their findings based on the respective study design (Greek and Menache, 2013). All the studies fulfilled less than half of the total score (7 out of 14) in their quality assessment, which is attributed to underreporting. Some of the missing information in the studies include the gender of the animal used, housing conditions, method of randomization, blinded assessment, power size calculation, and evidence of compliance with animal ethics. This information is frequently highlighted not only for ethical animal-handling purposes but also for ensuring the reproducibility of the *in vivo* test results. To establish the validity of the study design, a lack of standardization of the factors mentioned above would ultimately affect the reliability of the result, the values to which a conclusion can be drawn. Moreover, the data generated from good quality studies could be compared across other studies with higher confidence levels since a higher degree of standardization could be achieved.

Apart from animal experimental design, other missing information is related to nanoparticles’ characteristics, i.e., size, PDI, EE, and stability. Data assumption based on previous studies without validation effort was also observed (Conde et al., 2015; Fang et al., 2017; Li et al., 2018; Samiei Foroushani et al., 2019). Since nanomaterials are used as the test sample, changes to their properties would significantly impact the outcomes of the study. For instance, zeta potentials directly affect the physical stability of nanoparticles (How et al., 2011). Changes to this parameter due to sample degradation or contamination can indirectly impact the animal data. Moreover, when administered into the circulation, the surface charges of the NTA would attract different plasma proteins to form a protein corona via transient absorption (Bros et al., 2018). Consequently, depending on the binding strength and the overall size, the biodistribution and clearance of the NTA with its content will be altered. Failure to characterize the NTA’s property could eventually lead to standardization fallacy and nonreproducibility of results which further impact the clinical translation of animal research. The researchers, reviewers, and journal editors should emphasize the importance of standardization and critical parameter acquisition to improve the data reliability of animal studies (Martić-Kehl et al., 2012).

### 4.6 Limitations

This systematic review inherits the limitations of the originally included studies. The overall quality of the included studies was low, where the median quality score was only 6 out of the total 14. As all studies did not report on certain methodological details, specifically on the allocation concealment and blinded assessment of outcome, this could attribute to biases of findings. Given this, a cautious interpretation of the findings in this systematic review is warranted. Besides, the therapeutic performances of different NTAs were determined based on the reported doses that interfere with the tumor progression. Since the doses were acquired from different studies, a direct, definitive comparison across all the studies must be cautiously interpreted even though the same anticancer drug was used, in this case, 5FU. This is because tumor regression is a complex variable affected by treatment regimen, dosing frequency, duration, animal species, tumor location, and even cancer cell types. Apart from these biological factors, the nanoparticles’ size, charges, purity, preparation method, and operator biasness can further complicate data interpretation. When taken together, the alteration of 5FU’s PK and pharmacodynamics resulted in the variation of therapeutic and toxicity profiles. Moreover, the reported dosage in a few studies could only be arbitrarily normalized based on the molecular weight of nanomaterials due to the lack of drug EE information. In addition, there was no protocol registration for this systematic review. However, there was no other known protocol registered with a similar topic, and no major changes had been made to the methodology compared with the beginning of this systematic review.

All 5FU-NTAs were reported to have positive outcomes in terms of anti-tumor effect and tumor-imaging properties despite having variations in study design. Since a myriad of factors notably interferes with the outcome of the study, there may be a concern over selective reporting of positive results. Therefore, it is recommended that negative results should be highlighted as well.

### 5 Clinical Implications and Future Perspective

There has been a noticeable translation of nanomedicine from laboratory to various clinical uses. Since 1995, the success of Doxil^®^ has drawn the attention of more pharmaceutical companies to venture into nano-based products. For instance, Cristal Therapeutics has recently launched a Phase IIa clinical trial on CriPec^®^ Docetaxel in 2018 (NCT03742713), which was combined with Zirconium-89 for PET imaging. The nanoparticles evaluate the biodistribution and tumor accumulation to develop a better targeted therapy for ovarian cancer (Atrafi et al., 2019). Another company, Nanobiotix, has ventured into Phase I trials in 2020 for NBTXR3^®^ (hafnium oxide nanoparticles) as a radio-enhancer to curb soft-tissue sarcoma and pancreatic adenocarcinoma via radiation (NCT04484909) (Bonvalot et al., 2019).

A significant hurdle of cancer treatment is the intrinsic heterogeneities of tumors that greatly impact the anti-tumor response of cancer therapies, leaving patients vulnerable to the adverse effects of these treatments. The enhanced permeability and retention (EPR) effect differs substantially within (intratumoral heterogeneity) and between tumors (intratumoral heterogeneity), making it extremely difficult to predict the outcomes of nanomedicines (Golombek et al., 2018). Nanotheranostics, an emerging branch of nanomedicine, has tremendous promise in overcoming tumor heterogeneity due to its ability to visualize and monitor the biodistribution and accumulation of nanomedicines in tumors (Chen et al., 2017). The customizable functionality offered by nanotheranostics allows effective stratification of patients based on the EPR effects to enable the tailoring of treatment regimens for each patient to achieve personalized medicine (Golombek et al., 2018). A notable example is the use of ferumoxytol (iron oxide nanoparticles), initially approved for the treatment of iron-deficiency anemia, as an MRI contrast agent to quantify the EPR heterogeneity in tumors and predict patient response to nanoliposomal irinotecan (Onivyde) (Miller et al., 2015; Ramanathan et al., 2017).

Recently, a variety of on-demand near-infrared (NIR)-activated plasmonic NTA composed of metal nanomaterials (gold, platinum, palladium, copper, manganese, silver) had shown promising anticancer activity attributed to their photothermal and photodynamic properties. The metal nanoparticles efficiently convert light energy into highly localized heat energy (photothermal therapy) via a phenomenon known as localized surface plasmon resonance (LSPR) to destroy cancer cells and minimize collateral damage to adjacent normal tissues (He et al., 2021a; Cai et al., 2021; Shan et al., 2021; Shanmugam et al., 2021; Zhu et al., 2022). Plasmonic gold NTA-mediated photothermal therapy has also been tested in combination with chemotherapy and immunotherapy to achieve synergistic tumor inhibition in the 4T1 tumor model (Zhang et al., 2020; Chen et al., 2021). On the other hand, plasmonic NTA also possesses the ability to utilize light energy to generate reactive oxygen species (photodynamic therapy) for the destruction of tumors (He et al., 2021b; Chen et al., 2021; Shanmugam et al., 2021). In addition, plasmonic NTA enables real-time visualization of the tumor and precise tracking of NTA in the tumors via photoacoustic imaging to facilitate image-guided treatment (He et al., 2021a; Shan et al., 2021; Shanmugam et al., 2021; Zhu et al., 2022). These temporal and spatial information will provide guidance for physicians to optimize and tailor the treatment regimen for each patient to achieve personalized medicine.

Despite being an old drug with several known limitations, 5-fluorouracil remains an essential component of numerous chemotherapy regimens owing to its unique and lethal anticancer mechanism of action (Chalabi-Dchar et al., 2021). With the convergence of innovation in nanotheranostics, the true anticancer potential of 5-fluorouracil will be realized and potentially further extended to benefit other anticancer agents.

## 6 Conclusion

Nanotheranostic is still an emerging area under nanomedicine. Based on this review, innovative strategies such as chemo-photothermal therapy, magnetic-guided targeting, ligand-guided targeting, and NIR fluorescence imaging have been proposed. Although these advances are currently confined to laboratory settings, the idea to combine various treatment strategies with diagnostic tools into a single entity can be appealing in the management of cancers. This review illustrates the potential use of existing NTAs in preclinical stages by highlighting their toxicity, antitumor, and diagnostic properties. There are still many clinical challenges that need to be overcome before their utilities could be extended to practical applications. Toxicological data of NTA have not been substantially highlighted. The information about NTA’s clearance, tissue accumulation, and probe–drug interaction should be examined to explain or predict the potential adverse effects.

NTAs can be designed to accommodate various treatment strategies to curb cancers. Being submicron particles with basic passive-targeting properties, NTA can be formulated with homing ligands and superparamagnetic materials to deliver its content with better tumor specificity. Most of the research does not emphasize the safety and toxicity of the nanomaterials. With incomplete toxicological data provided, the therapeutic risk-benefit of the 5FU-NTA is still uncertain. Nevertheless, regardless of single or multi-modes, all 5FU-NTAs exhibited higher anti-tumor activity than 5FU alone. The 5FU-NTA that induced photothermal effect could achieve a complete tumor remission within the test period in mouse colon and melanoma cancer models. This effect was notably greater than that of 5FU-NTAs, which only performed tumor-targeting. The diagnostic ability of NTAs varies depending on the imaging technique used. In all instances, NTAs with targeting mechanisms are superior as they could rapidly concentrate in the tumor region. In summary, the NTA that is composed of tumor-targeting ligands and those which affect cancer via multiple mechanisms are a promising candidate for further development. Based on current NTA cancer research, we proposed a checklist of recommended parameters to be included for future NTA cancer studies.

## Data Availability

The original contributions presented in the study are included in the article/Supplementary Material, further inquiries can be directed to the corresponding authors.
